# A pilot study of alcohol screening and brief interventions in community pharmacies

**DOI:** 10.1186/1940-0640-7-S1-A22

**Published:** 2012-10-09

**Authors:** Niamh Fitzgerald, Derek Stewart, Mariesha Jaffray, Jackie Inch, Eilidh Duncan, Ebenezer Afolabi, Anne Ludbrook

**Affiliations:** 1 Create Consultancy, Ltd., Robert Gordon University, Schoolhill, Aberdeen, Scotland, UK; 2 School of Pharmacy and Life Sciences, Robert Gordon University, Schoolhill, Aberdeen, Scotland, UK; 3 Department of Mental Health, University of Aberdeen, Foresterhill, Aberdeen, Scotland, UK; 4 Center of Academic Primary Care, University of Aberdeen, Foresterhill, Aberdeen, Scotland, UK; 5 Health Services Research Unit, University of Aberdeen, Foresterhill, Aberdeen, Scotland, UK; 6 Health Economics Research Unit, University of Aberdeen, Foresterhill, Aberdeen, Scotland, UK

## 

No randomized controlled trials (RCTs) of screening and brief intervention (SBI) have been conducted in the community pharmacy setting. This pilot study was designed to inform the development and implementation of a large-scale RCT. The study examined the feasibility of providing SBI in community pharmacies, including practical considerations, recruitment of pharmacists and clients, uptake, potential effectiveness, and acceptability. A cluster RCT was conducted involving 20 community pharmacies. Pharmacy customers were screened using the Fast Alcohol Screening Test (FAST) to determine eligibility. The control group received a general lifestyle leaflet, while the intervention group was offered BI from a trained pharmacist. Clients in both groups were asked to complete baseline and three- and six-month postal questionnaires of self-reported alcohol consumption and to re-take the FAST. Qualitative work included follow-up telephone interviews with clients as well as focus groups with the public and participating pharmacists. Over 1000 clients were approached, with 77.6% (n = 846) completing the FAST. Of these, 27.1% (n = 229) were eligible for inclusion (FAST score = 3), 69 of whom (30.1%) consented to participate (27 in the intervention group and 42 in the control group). Nearly twice the number of eligible clients were recruited from control versus intervention pharmacies (41.6% versus 21.2%). A range of barriers and facilitators were identified by participating pharmacists and clients regarding SBI delivery. The main barrier for pharmacists was approaching clients for screening. Delivery of SBI was acceptable to most pharmacists and staff, however, future success of SBI in this setting will depend on identifying strategies for supporting practitioners in engaging pharmacy clients for screening.

